# Enterococcal Endocarditis in a Bicuspid Aortic Valve Associated With a Sinus of Valsalva Aneurysm

**DOI:** 10.7759/cureus.104214

**Published:** 2026-02-25

**Authors:** Naomi R Khanna, Alexa DenDulk, Adil Pervaiz

**Affiliations:** 1 Cardiology, Advanced Cardiology LLC, Hackettstown, USA; 2 Biomedical Engineering, Columbia University, New York, USA; 3 Cardiology, Morristown Medical Center, Morristown, USA

**Keywords:** bicuspid aortic valve, complete heart block, enterococcus faecalis, perivalvular abscess, sinus of valsalva aneurysm

## Abstract

This report describes a complex case of *Enterococcus faecalis* infective endocarditis (IE) in a 68-year-old man with an undiagnosed bicuspid aortic valve (BAV) and a concomitant sinus of Valsalva aneurysm (SoVA). A significant diagnostic challenge occurred as the patient’s initial febrile illness was misattributed to Lyme disease; two subsequent 30-day courses of doxycycline partially treated the infection, masking the clinical severity and delaying diagnosis. The patient eventually presented with new-onset atrial fibrillation, heart failure, and a new bifascicular block. Transesophageal echocardiogram (TEE) revealed a bulky vegetation on the BAV, leaflet perforation, and severe aortic regurgitation, alongside a perivalvular aortic root abscess. Despite the diagnostic delay and anatomical complexity, the patient was successfully managed with urgent aortic valve and root replacement (Bentall procedure) and the placement of a leadless pacemaker for postoperative complete heart block. This case illustrates the "perfect storm" of high-risk anatomy (BAV and SoVA) and antibiotic-induced diagnostic masking, emphasizing that new conduction abnormalities or heart failure in febrile patients must trigger immediate TEE to identify perivalvular extension.

## Introduction

Infective endocarditis (IE) involves the infection of the endocardial surface of the heart, most commonly affecting cardiac valves or intracardiac devices [1]. Key risk factors for native valve IE include structural cardiac abnormalities, particularly pre-existing valvular heart disease [1]. Specific anatomical substrates, such as bicuspid aortic valve (BAV) [2,3], and potentially a sinus of Valsalva aneurysm (SoVA) [2], create high-turbulence hemodynamic environments that predispose the endocardium to colonization. Among causative agents, *Enterococcus faecalis* is classified as a typical pathogen [1] and is being reported with increasing frequency [4,5].

The diagnosis of IE is rarely straightforward [6]. Identifying the infection is especially challenging when initial symptoms are nonspecific or "masked" by prior antimicrobial therapy, leading patients to present with advanced complications rather than classic signs of sepsis [6,7]. As seen in this case, a history of treatment for an unrelated febrile illness weeks prior to presentation is common. This clinical reality necessitates high vigilance, a focused initial evaluation, and diligent serial examinations [1,6].

IE is associated with a wide spectrum of complications, with cardiac involvement occurring in up to half of all cases [8]. Aortic root abscess is a rare but life-threatening complication [8-11] that requires early detection and prompt surgical intervention [1]. These abscesses, particularly in the setting of aortic valve involvement, often disrupt the cardiac conduction system, leading to heart block or bundle branch disturbances [9,11,12]. Additionally, new-onset atrial fibrillation (AFib) is common in severe infections [13] and serves as a poor prognostic marker associated with increased mortality in the setting of IE [14].

This case details an unusual presentation of enterococcal IE in a patient with a previously undiagnosed BAV and concomitant SoVA. The clinical significance lies in the "diagnostic masking" provided by prior doxycycline therapy, which led to a missed opportunity for early detection. The subsequent progression into a triad of severe complications, valvular destruction, aortic root abscess, and conduction abnormalities emphasizes the dangers of delayed diagnosis in patients with underlying risk factors and supports the use of early blood cultures and echocardiographic imaging in the evaluation of febrile patients [1].

## Case presentation

Presentation and initial evaluation

A 68-year-old man with a history of bladder cancer (status post-transurethral resection of bladder tumor (TURBT)) and chronic urinary retention requiring intermittent self-catheterization presented to the emergency department (ED) with a one-week history of progressive dyspnea and 24 hours of chest tightness. Notably, a little over 60 days prior to this admission, the patient developed a febrile illness. He was diagnosed with Lyme disease at an outside clinic and prescribed a 30-day course of doxycycline (100 mg twice daily). Despite this, he continued to have fevers and was prescribed a second 30-day course of doxycycline (100 mg twice daily), which he had completed 48 hours before presentation.

**Figure 1:**
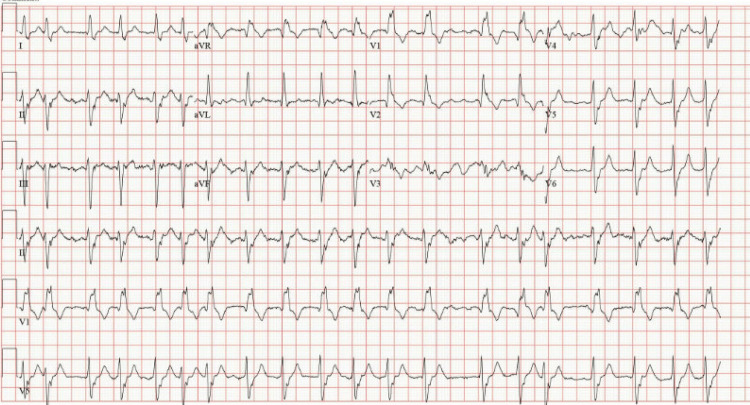
Electrocardiogram obtained in the emergency department demonstrating atrial fibrillation with rapid ventricular response and a new bifascicular block.

**Figure 2:**
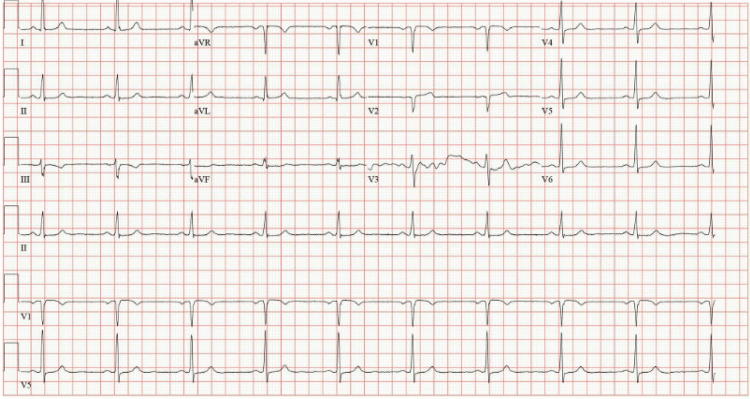
Prior electrocardiogram demonstrating normal sinus rhythm without evidence of bifascicular block.

**Figure 3:**
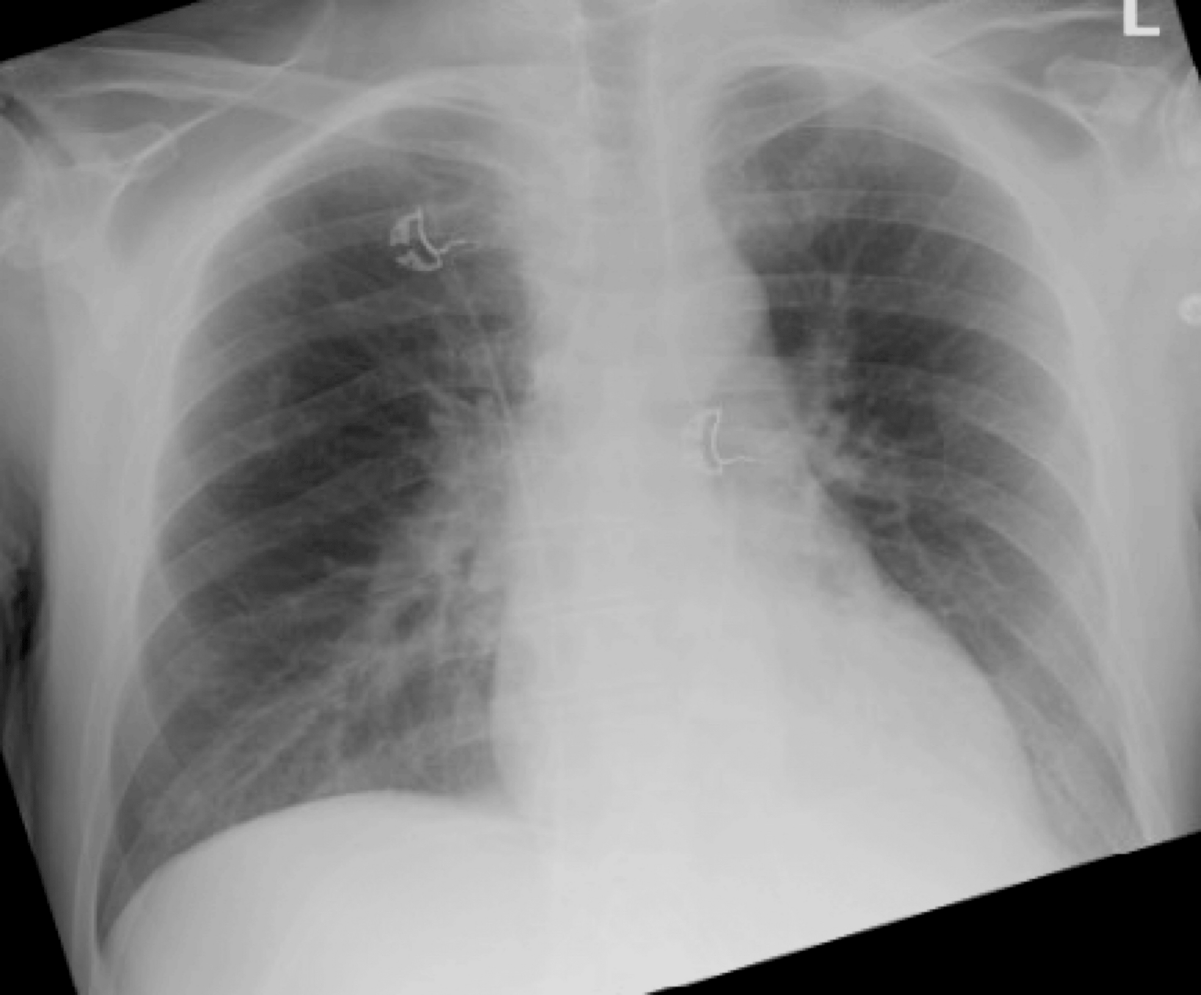
Chest radiograph (limited image quality) demonstrating pulmonary vascular cephalization, suggestive of congestive heart failure.

**Figure 4:**
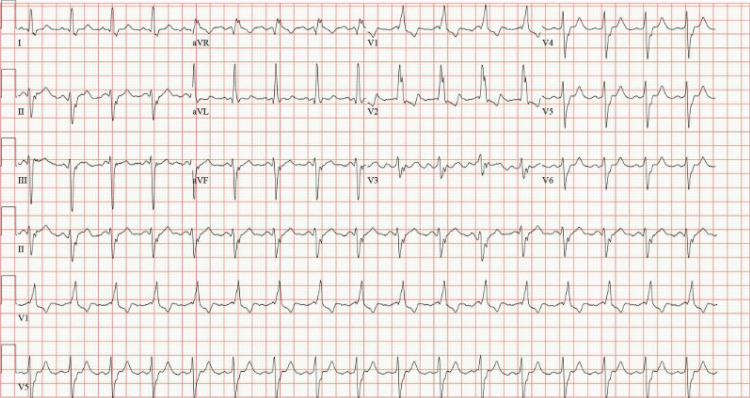
Follow-up electrocardiogram demonstrating sinus rhythm with persistent bifascicular block.

On initial physical examination, the patient was afebrile (98.2°F) but tachypneic. Chest auscultation revealed bilateral basilar crackles. Tachypnea and the irregular tachycardia made heart sounds difficult to discern. Jugular venous distension was noted at 10 cm, and trace bilateral pedal edema was present, consistent with acute heart failure. An initial electrocardiogram (ECG) revealed atrial fibrillation with rapid ventricular response and a new bifascicular block (Figure 1), a significant change from an ECG obtained one year prior (Figure 2). Initial laboratory studies were significant for an elevated white blood cell count of 12.8 thousand/L (normal 3.8-10.8) and a B-type natriuretic peptide (BNP) of 7342 pg/mL (normal < 900). The D-Dimer was 2.37 µg/mL (normal < 0.49) and CRP 96.6 mg/L (< 5). Chest radiography confirmed pulmonary vascular cephalization (Figure 3). The patient was initially managed for new-onset atrial fibrillation and heart failure secondary to rapid rate; he was started on beta-blockers and anticoagulation, subsequently converting to sinus rhythm with a persistent bifascicular block (Figure 4).

Clinical deterioration

On hospital day 2, the patient’s clinical status acutely worsened. He developed a fever of 102.4°F with rigors and increased oxygen requirements. Laboratory repeats showed a significant and acute drop in hemoglobin from 10.1 g/dL to 7.2 g/dL (Table 1).

**Table 1:** Lab values on presentation day 1 and day 2 WBC: white blood cell, NT-proBNP: N-terminal pro–B-type natriuretic peptide.

Lab value	Day 1	Day 2	Normal range
WBC	12.83	8	4.50-11.00 thousand/nL
Absolute neutrophils	11.49	6.69	2.00-6.60 thousand/nL
Neutrophils %	89	81	40.0%-80.0%
Hemoglobin	10.1	7.2	14.0-17.0 g/dL
Hematocrit	32.3	28.1	39.0%-50.0%
NT-proBNP	7242	-	<900 pg/mL

Diagnostic imaging and microbiology

**Figure 5:**
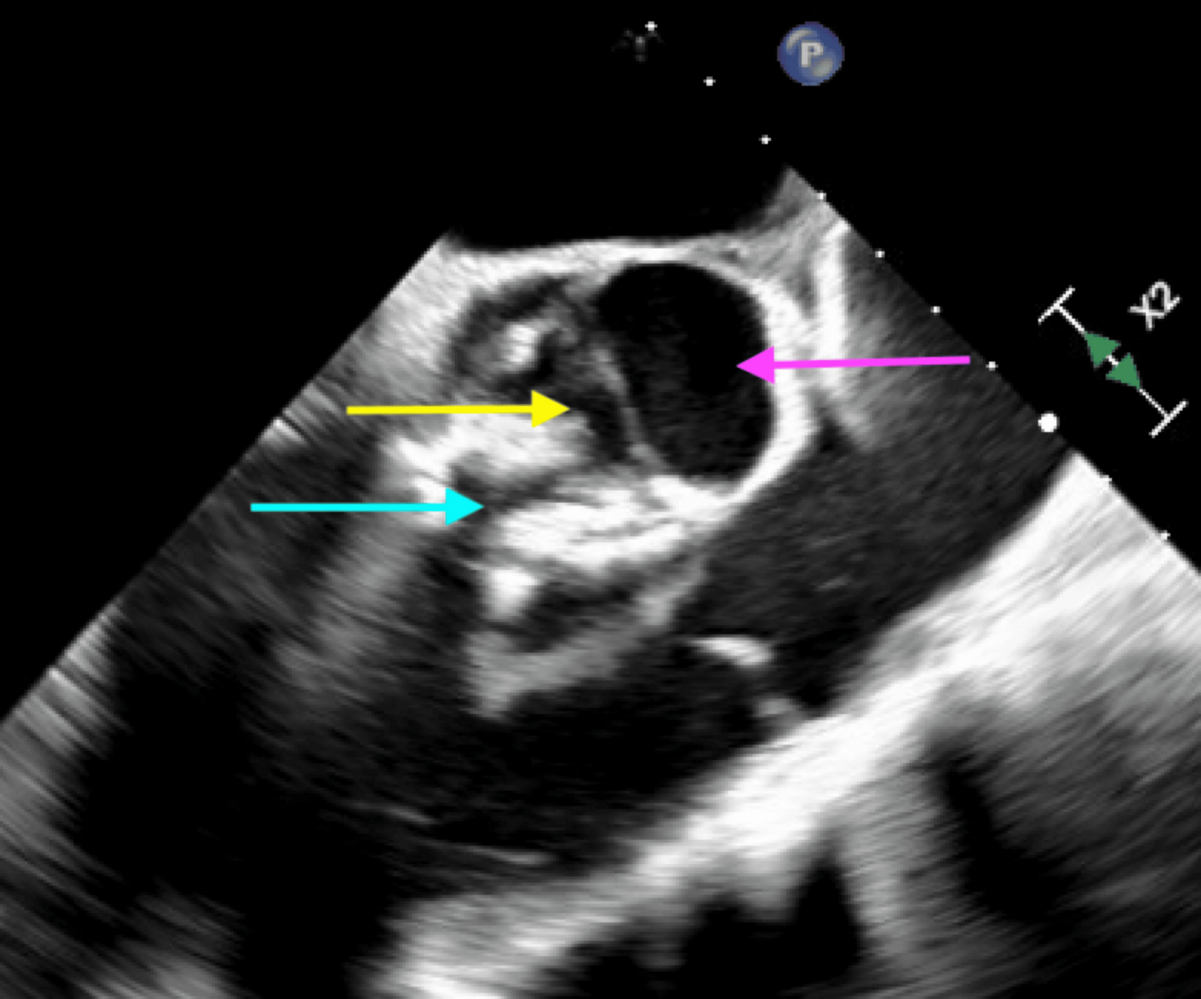
Transesophageal echocardiogram demonstrating a bicuspid aortic valve (yellow arrow). The pink and blue arrows indicate the left and right cusps, respectively.

**Figure 6:**
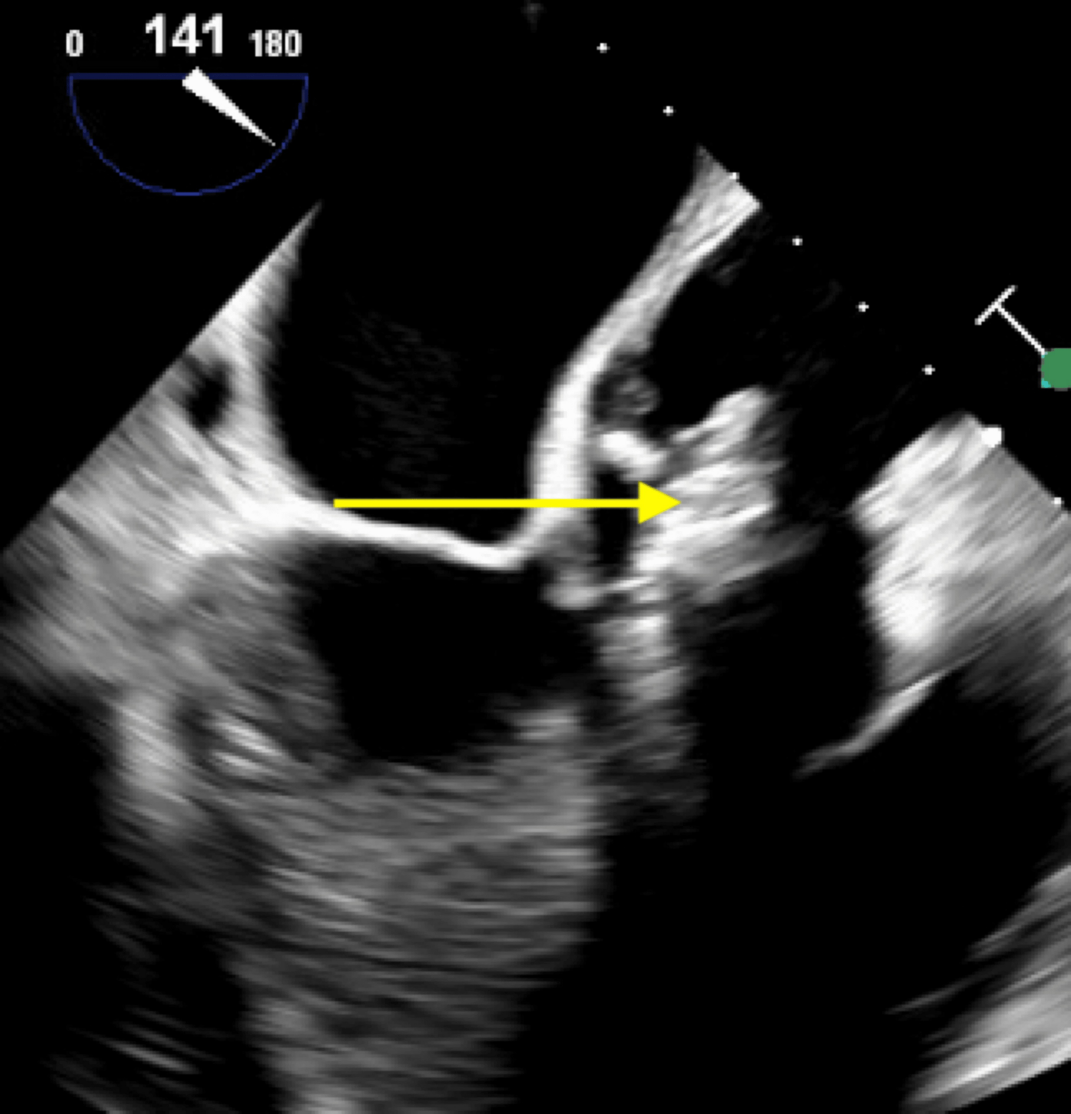
Transesophageal echocardiogram demonstrating a bulky, echodense lesion measuring 2.2 × 1.9 cm adherent to the right cusp (yellow arrow) (mid-esophageal long-axis (ME LAX) view).

**Figure 7:**
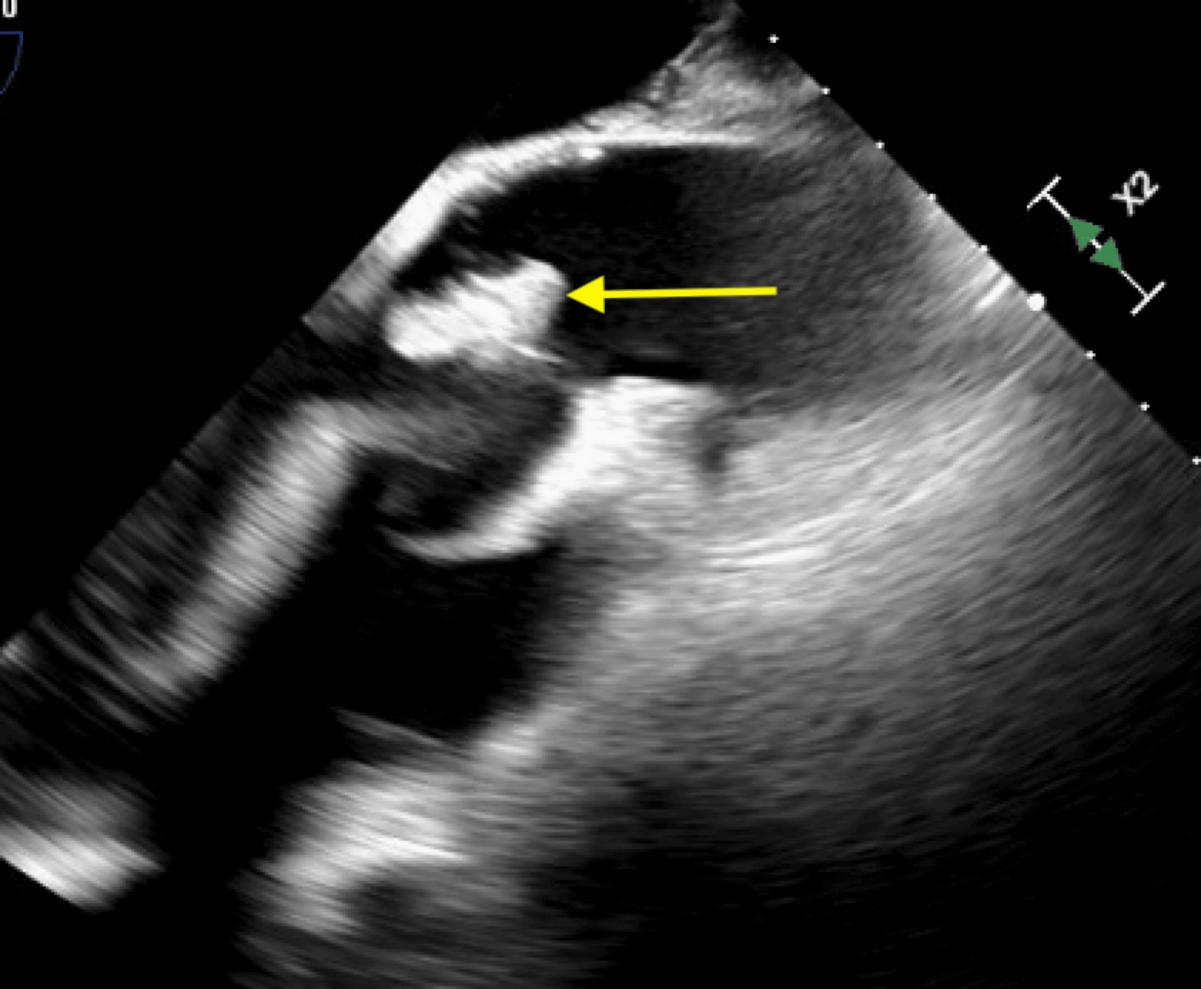
Transesophageal echocardiogram demonstrating a bulky, echodense lesion measuring 2.2 × 1.9 cm adherent to the right cusp (yellow arrow) (mid-esophageal aortic valve long-axis (ME AV LAX) view).

**Figure 8:**
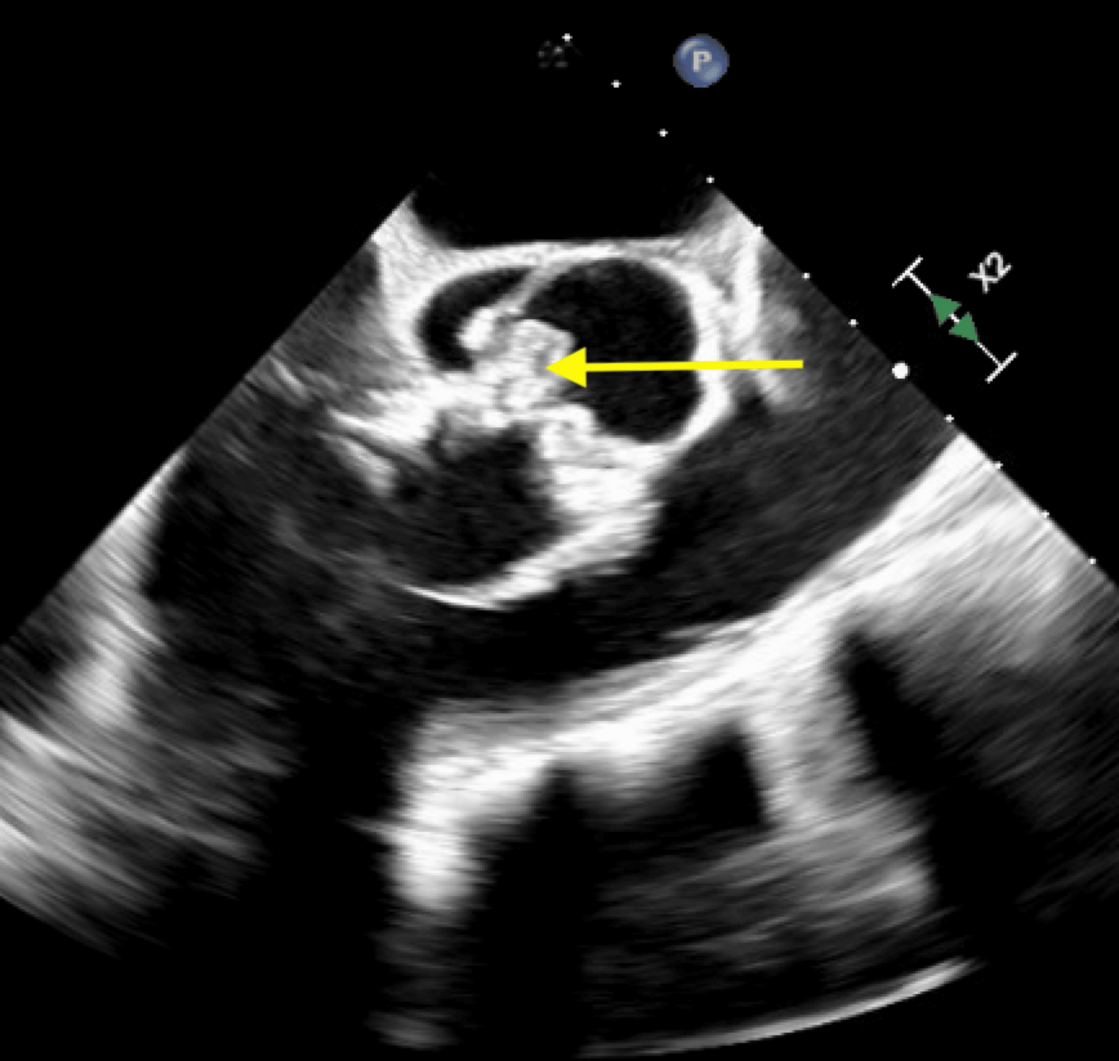
Transesophageal echocardiogram demonstrating a bulky, echodense lesion measuring 2.2 × 1.9 cm adherent to the right cusp (yellow arrow) (mid-esophageal aortic valve short-axis (ME AV SAX) view).

**Figure 9:**
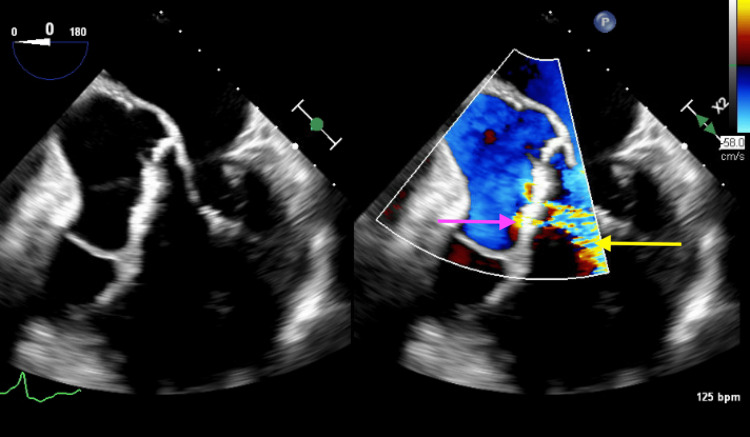
Transesophageal echocardiogram demonstrating perforation of the right cusp (pink arrow) with associated aortic regurgitation (yellow arrow).

**Figure 10:**
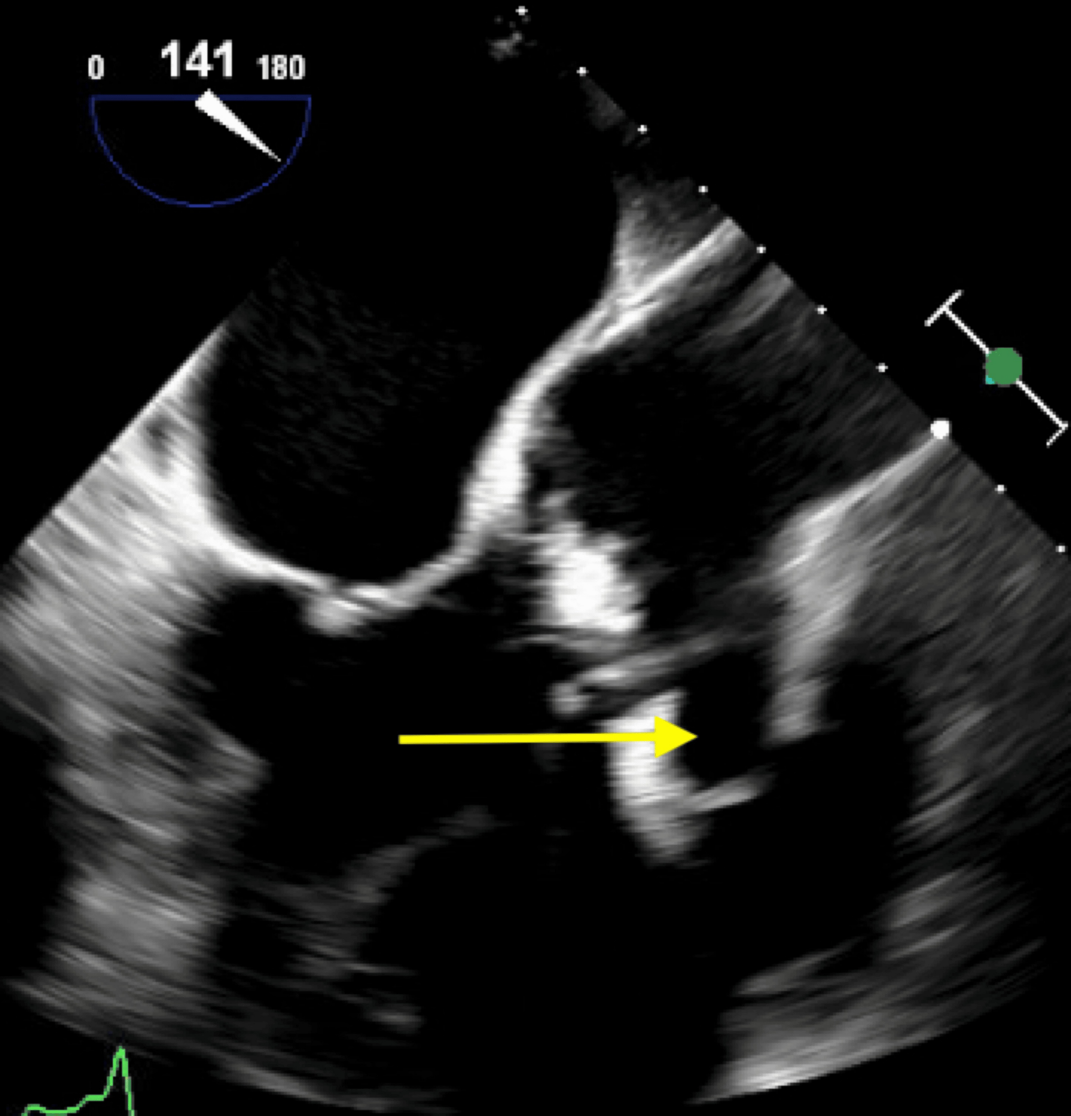
Transesophageal echocardiogram demonstrating a sinus of Valsalva aneurysm involving the right cusp (yellow arrow) (mid-esophageal long-axis (ME LAX) view).

**Figure 11:**
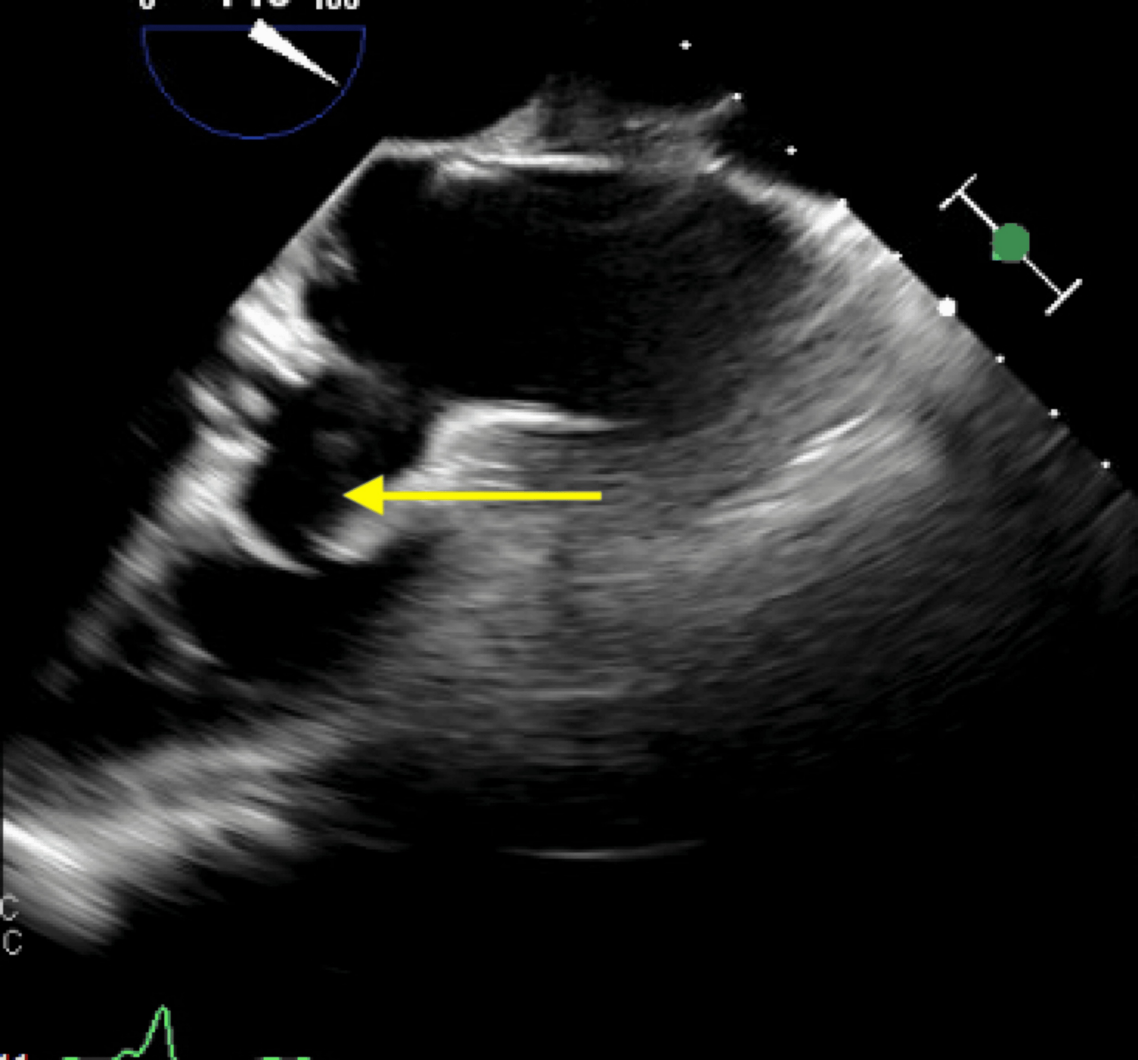
Transesophageal echocardiogram demonstrating a sinus of Valsalva aneurysm involving the right cusp (yellow arrow) (mid-esophageal aortic valve long-axis (ME AV LAX) view).

**Figure 12:**
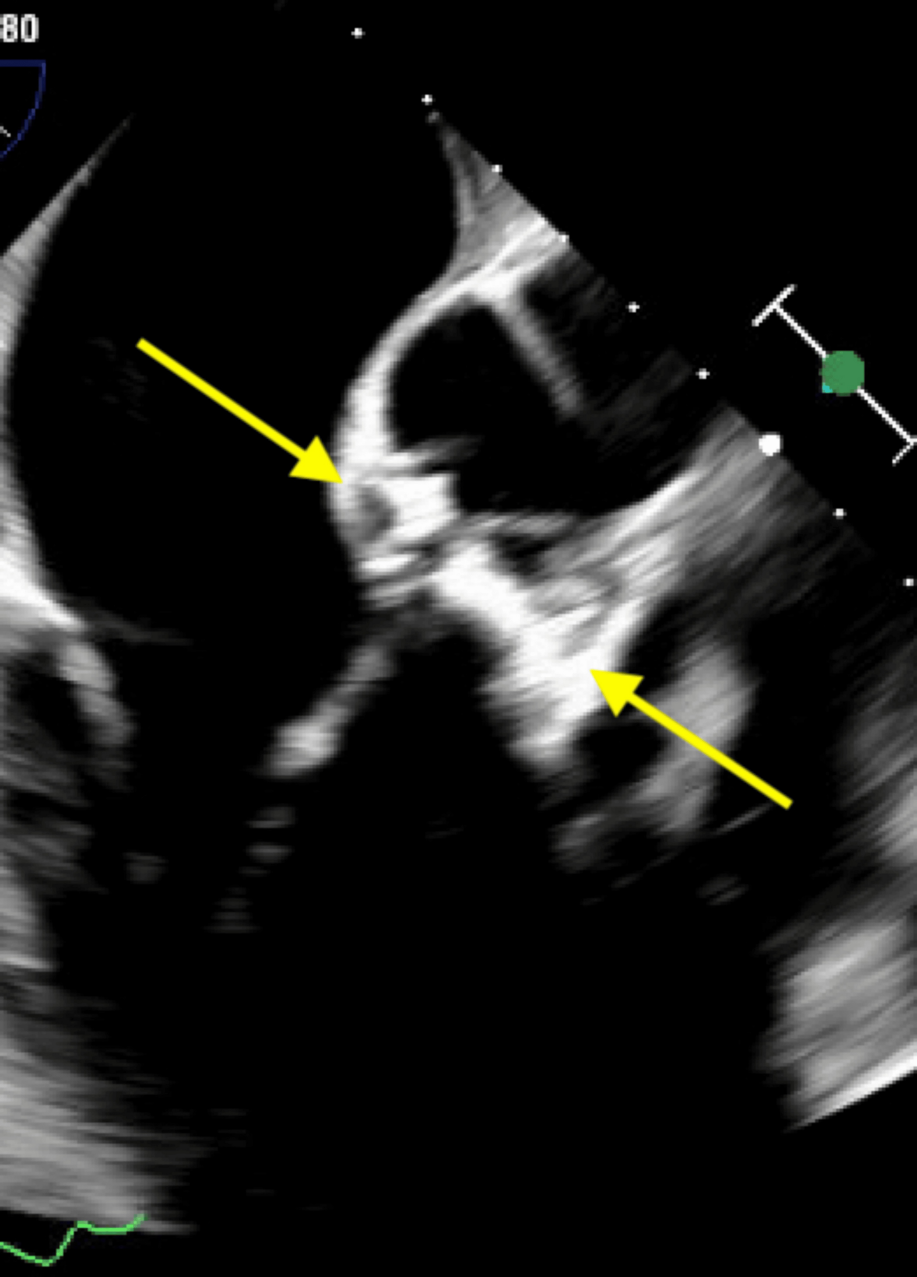
Transesophageal echocardiogram demonstrating an echodense, shaggy appearance of the aortic root, suggestive of an aortic root abscess (yellow arrow).

Blood cultures drawn in the ED (prior to the second day of fever) returned positive for *E. faecalis* in 2/2 sets. A transthoracic echocardiogram (TTE) followed by a transesophageal echocardiogram (TEE) was performed. The TEE revealed a BAV (Figure 5) with a large mobile, bulky vegetation measuring 2.2 × 1.9 cm that was seen adherent to the right coronary cusp (Figures 6-8). Findings included perforation of the right coronary cusp leaflet with severe aortic regurgitation (Figure 9) and aneurysmal dilation of the right sinus of Valsalva (Figures 10 and 11) with an associated perivalvular abscess (Figure 12).

Surgical intervention and postoperative course

The patient was emergently transferred for cardiac surgery. Intraoperative findings confirmed a BAV with a sinus of Valsalva aneurysm, a perforated right coronary cusp, endocarditis, and a circumferential annular abscess involving both the left and right coronary cusps. The native valve was excised, and the annulus was extensively debrided. A Bentall procedure was performed using a Valved Conduit Bovine #29 implant.

Postoperatively, the patient’s bifascicular block progressed to complete heart block. Due to the recent endocarditis and the risk of lead-related reinfection, a leadless pacemaker was implanted on postoperative day 7. Following a six-week course of intravenous ampicillin and ceftriaxone, the patient was discharged on postoperative day 25, hemodynamically stable, and without heart failure symptoms.

## Discussion

IE refers to infection of the endocardial surface of the heart, most commonly involving one or more cardiac valves or an intracardiac device [1]. An estimated 10,000 to 15,000 new cases of IE are diagnosed annually in the United States [15]. Pre-existing structural heart disease is present in approximately three-fourths of these cases [15]. Aortic valve involvement occurs in 12% to 30% of cases [15] and includes BAV, aortic stenosis, and aortic regurgitation [15]. While BAV is frequently associated with SoVA [2], it remains unclear whether the presence of a SoVA is an independent risk factor for IE [2]. In this case, the BAV likely provided the primary substrate for infection, while the SoVA potentially facilitated perivalvular extension. Additionally, it may not always be possible to distinguish between a congenital SoVA and one that develops secondary to infection-related tissue destruction or turbulent flow from aortic regurgitation [2].

The patient's history of chronic urinary retention and intermittent self-catheterization presents a plausible, though largely associative, portal of entry for bacteremia. *E. faecalis* is a common urinary tract colonizer, and enterococcal IE accounts for approximately 10% to 15% of IE cases [4,15]. Some reports cite prevalence rates as high as 26% [3], making it the third most common cause of endocarditis [3]. These patients often experience disproportionately high rates of complications and mortality.

The diagnosis of IE relies on a combination of clinical, microbiological, and imaging findings [1,3,6], with the 2023 Duke-International Society for Cardiovascular Infectious Disease criteria serving as the diagnostic standard [1]. However, classic findings may be absent, especially when "masked" by prior antibiotics like doxycycline, which can suppress bacteremia and delay the diagnosis [6]. TTE and TEE are central to the diagnosis [3]. TEE remains the preferred modality when IE or complications are suspected despite a negative TTE [1,6].

Atrial fibrillation is commonly observed in severe infections, and in the setting of IE, new-onset atrial fibrillation is a poor prognostic marker associated with increased mortality [14]. Cardiac complications occur frequently, with congestive heart failure (CHF) most often resulting from valvular regurgitation due to infection-induced structural damage [8]. Aortic valve IE is more likely to result in CHF compared to other valves [8].

Perivalvular abscess is a rare but life-threatening complication [6,9,10]. The aortic valve and its annulus are particularly susceptible [6,8], and BAV anatomy may carry a higher risk for perivalvular extension compared to tricuspid aortic valves [8]. Because of the proximity to the cardiac conduction system, conduction abnormalities, such as the bifascicular block seen in this patient, should prompt a strong suspicion for perivalvular abscess [6,8,11,16]. Mortality remains high, particularly when accompanied by moderate or severe valvular regurgitation [8].

TEE is significantly more sensitive than TTE for detecting these abscesses (90% vs. 43%) [6,8,9,10]. When findings are inconclusive, coronary computed tomography angiography (CCTA) may serve as an adjunct [6,9,10,12]. While antimicrobial therapy is the cornerstone of treatment, surgery is often necessary for patients with cardiac complications [1,6]. Surgery for left-sided IE is recommended (Class I) in the presence of CHF, heart block, or perivalvular abscess [6,7]. These complications carry high mortality, and surgery should not be delayed [1,11]. In this case, a leadless pacemaker was strategically utilized to manage heart block while minimizing the risk of secondary device infection in a recently infected field.

## Conclusions

This case highlights the critical diagnostic challenge posed by "masked" IE, where prior antibiotic therapy for unrelated diagnoses can suppress clinical symptoms while perivalvular destruction progresses. The presence of a BAV and SoVA created a high-risk anatomical substrate, but the most vital clinical "red flag" was the development of new conduction abnormalities. These findings should immediately trigger TEE to evaluate for perivalvular abscess, regardless of prior negative cultures. Ultimately, the successful management of complex *E. faecalis* IE hinges on early recognition, adherence to surgical guidelines for early intervention, and the strategic use of technologies, such as leadless pacemakers when needed, to mitigate postoperative infection risks.
